# Ethyl Acetate Extract of *Scindapsus* cf. *hederaceus* Exerts the Inhibitory Bioactivity on Human Non-Small Cell Lung Cancer Cells through Modulating ER Stress

**DOI:** 10.3390/ijms19071832

**Published:** 2018-06-21

**Authors:** Chon-Kit Chou, Wangta Liu, Yu-Jie Hong, Hans-Uwe Dahms, Chen-Hao Chiu, Wen-Tsan Chang, Ching-Ming Chien, Chia-Hung Yen, Yuan-Bin Cheng, Chien-Chih Chiu

**Affiliations:** 1 Graduate Institute of Natural Products, Kaohsiung Medical University, Kaohsiung 807, Taiwan; fatchou1988@hotmail.com (C.-K.C.); chyen@kmu.edu.tw (C.-H.Y.); 2 Department of Biotechnology, Kaohsiung Medical University, Kaohsiung 807, Taiwan; liuwangta@kmu.edu.tw (W.L.); reficul850429@gmail.com (Y.-J.H.); plusbeetle10@gmail.com (C.-H.C.); willness812@gmail.com (C.-M.C.); 3 Department of Biomedical Science and Environmental Biology, Kaohsiung Medical University, Kaohsiung 807, Taiwan; hansd@kmu.edu.tw; 4 Division of General and Digestive Surgery, Department of Surgery, Kaohsiung Medical University Hospital, Kaohsiung 807, Taiwan; wtchang@kmu.edu.tw; 5 Department of Surgery, School of Medicine, College of Medicine, Kaohsiung Medical University, Kaohsiung 807, Taiwan; 6 Center for Infectious Disease and Cancer Research, Kaohsiung Medical University, Kaohsiung 807, Taiwan; 7 Department of Medical Research, Kaohsiung Medical University Hospital, Kaohsiung 807, Taiwan; 8 Research Center for Environment Medicine, Kaohsiung Medical University, Kaohsiung 807, Taiwan; 9 Center for Stem Cell Department of Biological Sciences, National Sun Yat-sen University, Kaohsiung 804, Taiwan; 10 Translational Research Center, Cancer Center and Department of Medical Research, Kaohsiung Medical University Hospital, Kaohsiung 807, Taiwan; 11 The Graduate Institute of Medicine, Kaohsiung Medical University, Kaohsiung 807, Taiwan

**Keywords:** *Scindapsus* cf. *hederaceus*, ethyl acetate extract, unfolded protein response, UPR, non-small cell lung cancer cell, NSCLC, ER stress, selective anti-cancer therapeutics

## Abstract

Unfolded protein response (UPR) is a cytoprotective mechanism that alleviates the protein-folding burden in eukaryotic organisms. Moderate activation of UPR is required for maintaining endoplasmic reticulum (ER) homeostasis and profoundly contributes to tumorigenesis. Defects in UPR signaling are implicated in the attenuation of various malignant phenotypes including cell proliferation, migration, and invasion, as well as angiogenesis. This suggests UPR as a promising target in cancer therapy. The pharmacological effects of the plant *Scindapsus* cf. *hederaceus* on human cancer cell lines is not understood. In this study, we identified an ethyl acetate extract from *Scindapsus* cf. *hederaceus* (SH-EAE), which markedly altered the protein expression of UPR-related genes in human non-small cell lung cancer (NSCLC) cells. Treatment with the SH-EAE led to the dose-dependent suppression of colony forming ability of both H1299 and H460 cells, but not markedly in normal bronchial epithelial BEAS-2B cells. SH-EAE treatment also attenuated the migration and invasion ability of H1299 and H460 cells. Moreover, SH-EAE strikingly suppressed the protein expression of two ER stress sensors, including inositol requiring enzyme-1α (IRE-1α) and protein kinase R-like ER kinase (PERK), and antagonized the induction of C/EBP homologous protein (CHOP) expression by thapsigargin, an ER stress inducer. SH-EAE induced the formation of massive vacuoles which are probably derived from ER. Importantly, SH-EAE impaired the formation of intersegmental vessels (ISV) in zebrafish larvae, an index of angiogenesis, but had no apparent effect on the rate of larval development. Together, our findings demonstrate, for the first time, that the ability of SH-EAE specifically targets the two sensors of UPR, with significant anti-proliferation and anti-migration activities as a crude extract in human NSCLC cells. Our finding also indicates potential applications of SH-EAE in preventing UPR activation in response to Tg-induced ER stress. We suggest that SH-EAE attenuates UPR adaptive pathways for rendering the NSCLC cells intolerant to ER stress.

## 1. Introduction

Accumulation of misfolded or unfolded proteins in the ER lumen is a feature common to all malignant tumors during the multi-step progression from hyperplastic lesions. The occurrence of endoplasmic reticulum (ER) stress has been detected in about 55.9% to 87.5% of human non-small cell lung cancer (NSCLC) tissue samples [1]. It takes place in mammalian cells as a result of nutrient deprivation, hypoxia, oxidative stress, aberrant glycosylation status, and loss of calcium homeostasis [2]. Effective alleviation of such protein-folding burden is essential for maintaining ER homeostasis, and is primarily achieved by unfolded protein response (UPR), which is an evolutionarily conserved signaling pathway in eukaryotic cells [3]. Notably, NSCLC cells with high proliferation rates and chemoresistant characteristics depend on the activation of UPR [2]. Accumulating evidence also suggests that an increase of UPR-related proteins could serve as a prognostic predictor and a candidate for targeted therapy [4,5,6,7,8,9,10]. However, the mechanisms by which the UPR mediates the tumorigenesis and NSCLC progression are complex, multifactorial, and remain to be elucidated.

During ER stress, the bulk of misfolded or unfolded proteins accumulate in the lumen of ER. To cope with ER stress, there are three ER transmembrane sensors responsible for triggering UPR, including activation of transcription factor 6 (ATF6), inositol-requiring enzyme 1α (IRE-1α), and protein kinase R-like ER kinase (PERK) [11]. Under normal conditions, the luminal domains of these sensors are occupied by the ER chaperone glucose-regulated protein 78 (GRP78), which keeps these three sensors in an inactive state. Upon ER shifting from normal to a stress condition, GRP78 releases from the ER stress sensors and binds instead to the unfolded proteins, which triggers these sensors to follow three fundamentally different but complementary signaling cascades. Briefly, ATF6 translocates from the ER to the Golgi apparatus where it is cleaved to yield the active cytosolic fragment of ATF6, which acts as a transcription factor to activate the transcription of chaperones [12,13]; PERK is a transmembrane kinase that phosphorylates α-subunit of eukaryotic initiation factor 2 (eIF2α) and then propagates to downstream proteins including activating transcription factor 4 (ATF4) and C/EBP homologous protein (CHOP), thereby initiating a global translational reduction [14]; IRE-1α undergoes the oligomerization and performs its endoribonuclease activity to splice out of the 26-nucleotide intron from the mRNA encoding X-box binding protein 1 (XBP-1). The mature form of XBP-1, acting as a transcription activator, further induces the transcription of genes that participate in protein folding and ER-associated degradation [15].

Promoting basal levels of UPR in various cancer models promotes tumor cell growth and migration, develops chemoresistance, and adapts to harsh environments [2,16]. When UPR is hyperactivated but also unable to restore the ER homeostasis, the UPR signaling can be switched from prosurvival to proapoptotic, leading to massive cell death [17,18]. Such phenomena have prompted many researchers to identify ER stress-inducing drugs against a wide range of cancers. Several related agents have been identified, including tunicamycin (Tm) and thapsigargin (Tg). However, such attempts have either led to significant adverse reactions or were not developed further for clinical use, probably due to the lack of specificity and the incidence of systemic toxicity [19]. Tm is a nucleoside antibiotic frequently used to study ER stress. This agent inhibits the transferase responsible for adding *N*-acetylglucosamine-1-phosphate from sugar nucleotide to dolichyl phosphate which results in the inhibition of protein glycosylation and severe ER stress [20]. However, potential clinical use of tunicamycin is not permitted, primarily due to its nephrotoxicity [19]. Tg, for example, is a potent candidate to trigger UPR in different types of cancer. However, Tg enhances ER stress by blocking the sarcoplasmic or endoplasmic reticulum Ca^2+^-ATPase family of calcium pumps, which in turn causes the leakage of calcium out of the ER storage compartment [21]. Perturbation of calcium flux is certainly not feasible in medical applications because calcium is implicated in several physiological functions [22]. Hence, there is an urgent demand to identify novel ER stress-inducing agents, preferably with mild toxicity, for treating various types of cancer. In addition, although the activation of UPR could switch from cytoprotective to apoptosis, the signaling outputs of UPR are more targeting survival. Thus, if the cancer cells are only exposed to exogenous ER stress, it would increase the risk of the cancer cells to acquire a more robust UPR capacity. Hence, suppressing the UPR pathway appears to be a promising strategy for blocking the adaptive response to ER stress evoked in a wide range of cancers.

*Scindapsus* cf. *hederaceus* is a flowering plant belonging to the family Araceae. This genus includes 35 accepted species (www.theplantlist.org), and the species in this genus are mainly distributed in the northeastern India to western Polynesia. Only few of them have been biologically or pharmacologically investigated. Among them, *Scindapsus officinalis* is widely used in Indian ethnomedicine for the treatment of skin diseases and asthma [23]. The fruit from *Scindapsus officinalis*, which has been reported to treat various human ailments, is the only plant part of this genus subjected to previous phytochemical investigation [24]. However, relevant research on other species, e.g., *Scindapsus* cf. *hederaceus*, are rare. To reveal the therapeutic potential of this species, our research will focus on mechanistic studies of the anti-carcinogenic effects of the extract from *Scindapsus* cf. *hederaceus*.

## 2. Results

### 2.1. Functional Screening to Identify Crude Extract as a UPR Targeting from Plants in the Family Araceae

**Figure 1:**
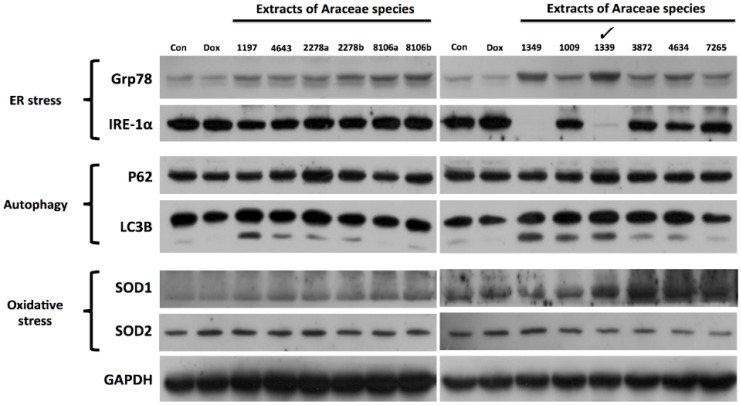
Identification of ethyl acetate extract of *Scindapsus* cf. *hederaceus* as a novel UPR modulator. The 12 samples of 10 plant species, labeled as 1197 (ethyl acetate), 4643 (ethyl acetate), 2278a (ethyl acetate), 2278b (water), 8106a (water), 8106b (butanol), 1349 (methanol), 1009 (ethyl acetate), 1339 (ethyl acetate), 3872 (ethyl acetate), 4634 (hexane), and 7265 (ethyl acetate), were collected from Dr. Cecilia Koo Botanic Conservation Center, Kaohsiung County, Taiwan. Ethyl acetate extract of *Scindapsus* cf. *hederaceus* (SH-EAE) was labeled as 1339 and stored at −20 °C for the screening of biological activity. H1299 cells were exposed to a single dose (20 μg/mL) of 12 extracts from a family Araceae for 48 h followed by immunoblot assay. The protein levels of Grp78, IRE-1α, SQSTM1, LC3, SOD1, and SOD2 were evaluated. Dimethyl sulfoxide (DMSO) as vehicle control. Dox, Doxorubicin. Glyceraldehyde-3-phosphate dehydrogenase (GAPDH) was used as the loading control.

A severe response to ER stress frequently leads to the catastrophic death of the affected cell. To avoid this harmful hyperactivation of the ER stress signaling, a milder way of inducing ER stress essentially needs to be developed, thus helping us to better understand the adaptive mechanism known as UPR to cope with ER stress. In this study, we screened a subset of plant extracts from a family Araceae for their ability to target specific UPR components. One of these candidates was isolated from an ethyl acetate extract of *Scindapsus* cf. *hederaceus* (its identification number in the library is 1339), which we labeled as “SH-EAE.” Applied at a concentration of 20 μg/mL, SH-EAE increased the protein expression of the UPR regulator Grp78, while it decreased the expression of IRE-1α (Figure 1), which is one of the three major ER stress sensors. So far, this alteration appears to be specific because of SH-EAE slightly but not significantly altered the protein expression of other pathway markers, including autophagy markers: P62/SQSTM1 (sequestosome 1) and LC3 (microtubule-associated protein 1A/1B-light chain 3), as well as free radical metabolism markers: SOD1 (superoxide dismutase 1) and SOD2 (superoxide dismutase 2) (Figure 1).

### 2.2. SH-EAE Altered the Key Regulators of Unfolded Protein Response (UPR)

**Figure 2:**
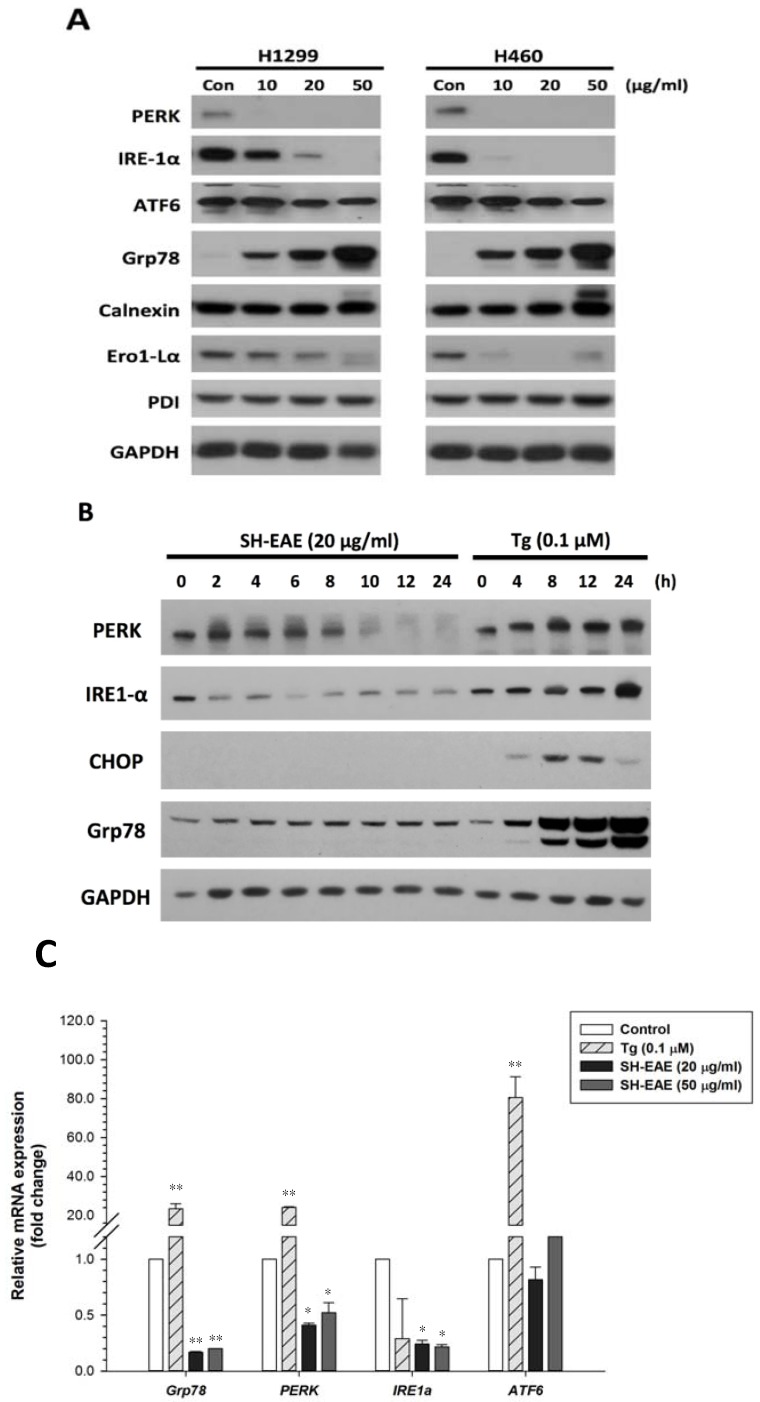

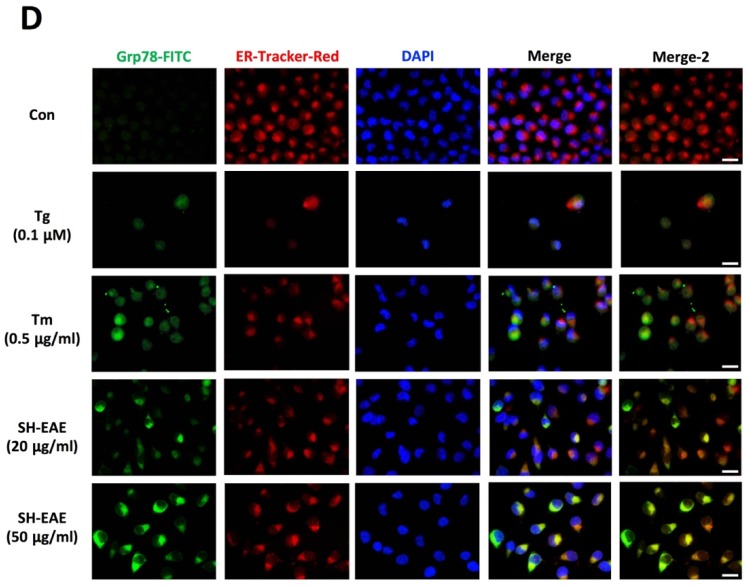
SH-EAE decreased both protein and mRNA expression of two ER stress sensors, PERK and IRE-1α. (**A**) H1299 and H460 cells treated with different concentrations of SH-EAE (10, 20, and 50 μg/mL) for 48 h. Protein lysates were collected and subjected to SDS-PAGE followed by immunoblotting using antibodies against Grp78, IRE-1α, PERK, ATF6, Calnexin, PDI, Ero-Lα, and GAPDH (loading control). (**B**) H1299 cells were treated with 20 μg/mL SH-EAE for 0, 2, 4, 6, 8, 10, 12, and 24 h. Protein lysates were subjected to SDS-PAGE followed by immunoblotting using antibodies against PERK, IRE-1α, CHOP, and Grp78. GAPDH served as the loading control. (**C**) Relative fold change of mRNA abundance of UPR-related genes including *GRP78*, *PERK*, *IRE1α*, and *ATF6*, was measured by qPCR in NSCLC H1299 cells treated with SH-EAE (20 and 50 μg/mL) or Tg (0.1 μΜ) for 24 h. Data are shown as fold change compared with vehicle-treated cells, and represent the mean ± SD of three independent experiments. (* *p* < 0.05; ** *p* < 0.001). (**D**) SH-EAE causes the accumulation of the Grp78 within the ER. Immunofluorescence staining of endogenous Grp78 proteins. H1299 cells were treated for 48 h with SH-EAE (20 and 50 μg/mL), Tg (0.1 μΜ), and Tm (0.5 μg/mL), respectively, followed by immunofluorescence staining using anti-Grp78 antibody and ER-Tracker Red. The nuclei were counterstained with 4’6-diamidino-2-phenylindole (DAPI). Scale bar indicates 50 μm. Representative images of three independent experiments are shown.

To further confirm whether SH-EAE is an inducer of the ER stress in NSCLC cells, we investigated the markers of UPR in NSCLC cells. As shown in Figure 2A, protein expression of PERK, IRE-1α, ATF6, Grp78, Ero1-Lα, PDI, and Calnexin were determined in both H1299 and H460 cells after 48 h of treatment with 10, 20, and 50 μg/mL SH-EAE. Among them, PERK, IRE-1α, and Ero1-Lα were markedly downregulated in a dose-dependent manner, while Grp78 expression was gradually upregulated in both cell lines. In addition, the initial appearance of ER stress response in these SH-EAE-treated cells was shown by the mild induction of Grp78 at around 2–4 h, which was maintained at a constant level over the following 6 to 24 h (Figure 2B). There was also a sharp decrease in the protein expression of ER stress sensors, IRE-1α and PERK, after 2 and 10 h, respectively. This data implies that the adaptive response (UPR) of NSCLC cells to ER stress is partly compromised by SH-EAE, which might reduce the resilience of cells against ER stress. Besides, we further assessed whether SH-EAE alters the mRNA levels of Grp78 as well as the three UPR sensors-PERK, IRE-1α, and ATF6. RT-qPCR was used to measure the relative change in mRNA expression after treatment of H1299 cells with two different doses of SH-EAE (20 and 50 μg/mL) or Tg (0.1 μM). The results showed that the levels of *GRP78*, *PERK*, and *IRE1α* mRNA were decreased significantly after treatment with SH-EAE for 24 h (Figure 2C). In contrast, the UPR activator, Tg caused a robust increase in Grp78, PERK, and ATF6 mRNA expression, suggesting that the UPR activation is truly elicited by Tg but is not elicited by SH-EAE. Besides, a decrease in Grp78 mRNA level was highlighted in this cell line after treatment with SH-EAE (Figure 2C), while a mild increase in its protein expression was also noticed at the same time point (Figure 2B). These data reflect a mechanism by which SH-EAE may modulate Grp78 at both the transcriptional and translational levels. Next, we performed immunofluorescence assays to monitor the cellular localization of Grp78, which has been implicated in the folding and maturation of proteins in ER and is raised dramatically under ER stress conditions [25]. As shown in Figure 2D, SH-EAE treatment led to increased amounts of Grp78 per cell in a dose-dependent manner. Notably, SH-EAE-treated cells exhibited a significant overlap between Grp78 and ER-Tracker Red, indicating that SH-EAE-induced stress that increases the concentration of Grp78 proteins are mainly confined to the ER.

### 2.3. Induction of Cytoplasmic Vacuoles in SH-EAE-Treated Cells

**Figure 3:**
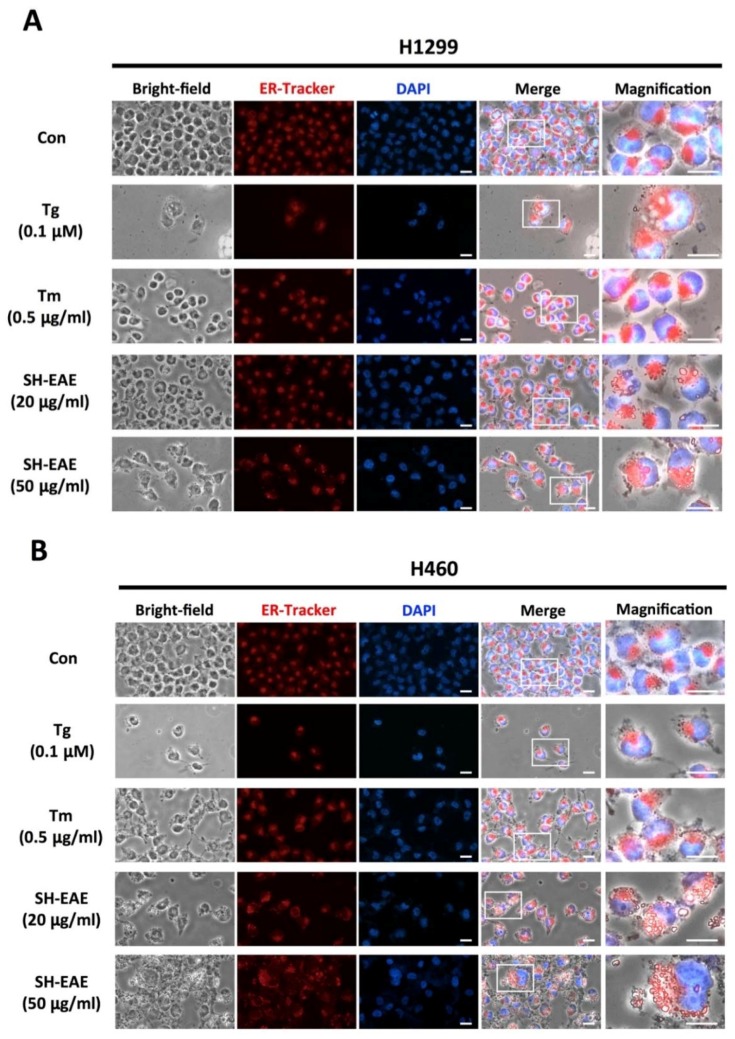

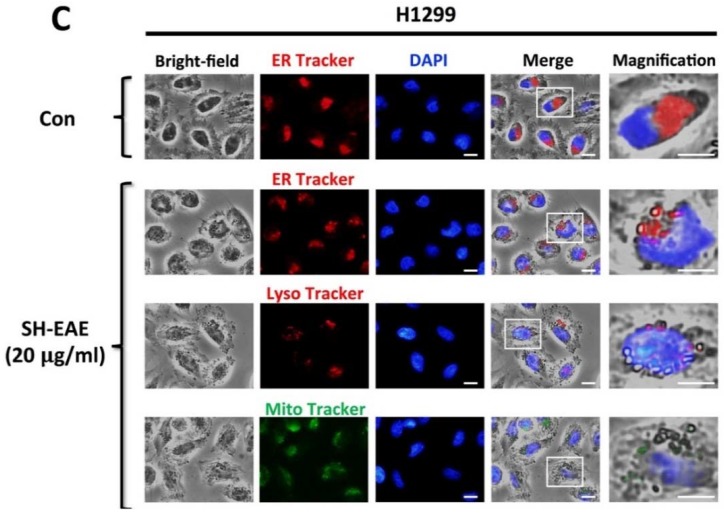
SH-EAE induced cytoplasmic vacuolization in NSCLC cells. Comparison of ER morphology affected by SH-EAE, Tg or Tm. (**A**) H1299 and (**B**) H460 cells expressing the fluorescent ER-Tracker were treated with SH-EAE (20 μg/mL), Tg (0.1 μM), or Tm (0.5 μg/mL) for 48 h and analyzed by Nikon Eclipse TE2000U inverted fluorescence microscope (Nikon Corporation, Tokyo, Japan). The images on the left panel were taken with a bright-field objective. The second panel is that stained with ER-Tracker™ Red dye. The third panel is that stained with DAPI. The fourth panel is an overlay of the bright-field images with the fluorescence images. The last panel is a magnification of the boxed region. Scale bar indicates 50 μm. Representative images of H1299 and H460 cells for each condition over three independent experiments are shown. (**C**) Staining for various organelles in the SH-EAE-treated H1299 cells. Cells exhibiting SH-EAE-induced vacuolization were stained for trackers of ER (red), lysosomes (red), and mitochondria (green). Cell nuclei were stained with DAPI. Scale bar indicates 25 μm. None of the lysosome or mitochondria trackers overlapped with SH-EAE-induced vacuolization.

To examine more precisely the impact of SH-EAE on ER, we used immunofluorescence microscopy to study SH-EAE-treated NSCLC cells. The ER membrane of cells was labeled with ER-Tracker Red, a dye for selectively staining the potassium channels enriched in ER. Two classical ER stress inducers, tunicamycin (Tm) and thapsigargin (Tg), served as positive controls. When NSCLC cell lines H1299 and H460 were treated with SH-EAE at concentrations of 20 μg/mL, both cell lines showed massive vacuolization in the vicinity of the nucleus (Figure 3A,B). However, these results also suggest that vacuoles are likely derived from ER as the regions where cytoplasmic vacuoles were significantly overlapping with ER-Tracker Red signals. In contrast, little to no vacuole formation appeared in both H1299 and H460 cells treated with Tg or Tm. Their red fluorescent signals enable visualization of the typical structure of the ER, which originates from the nuclear envelope and radiates toward the periphery, similar to what was seen in control cells. To further check the relationship between SH-EAE-induced vacuoles and ER, we stained with specific markers for several organelles. Following treatment with SH-EAE, vacuoles observed using phase-contrast microscopy were selectively colocalized with the ER-Tracker Red, but not stained with the trackers for lysosomes or mitochondria (Figure 3C). Altogether, these results indicate that cytoplasmic vacuoles induced by SH-EAE might originate from the ER. The induction of ER stress resembles the phenotype called paraptosis [26].

### 2.4. SH-EAE Selectively Decreased Colony-Forming Ability in NSCLC Cells

**Figure 4:**
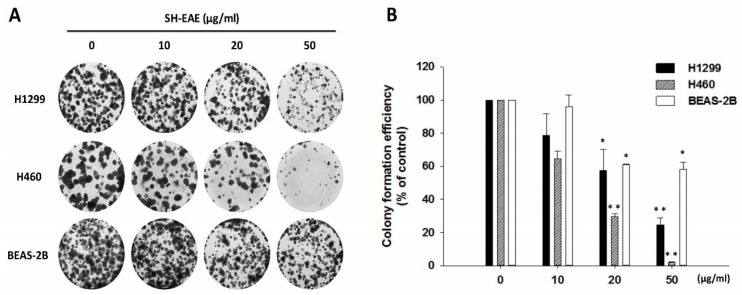
SH-EAE treatment inhibited colony-forming ability in malignant NSCLC cells but not in normal human bronchial epithelial cells. (**A**) Colony formation assay in 6-well plates. Two NSCLC cell lines, H1299 and H460, and non-tumorigenic bronchial epithelial BEAS-2B cells were treated with different concentrations of SH-EAE (10, 20, and 50 μg/mL) for 14 days. Afterward, the cells were fixed in 4% paraformaldehyde and stained with Giemsa. (**B**) The quantification analysis of the colony area. Cells were treated as described above, bright-field images were obtained independently with the same objective lens and the areas covered by the cell colonies were measured using ImageJ software. Data represent the mean ± SD of three independent experiments (* *p* < 0.05; ** *p* < 0.001).

Since physiological levels of ER stress are critical for regulating the malignant behaviors of cancer cells, we examined the colony-forming ability of two NSCLC cell lines H1299 and H460 and a non-tumorigenic human bronchial epithelial cell line BEAS-2B after 14 days of SH-EAE treatment. In this setting, SH-EAE effectively attenuated the colony-forming ability of NSCLC cells in a dose-dependent manner, whereas the non-tumorigenic BEAS-2B cells exhibited less sensitivity to SH-EAE (Figure 4A,B).

### 2.5. SH-EAE Reduced the Migration and Invasive Ability of NSCLC Cells

**Figure 5:**
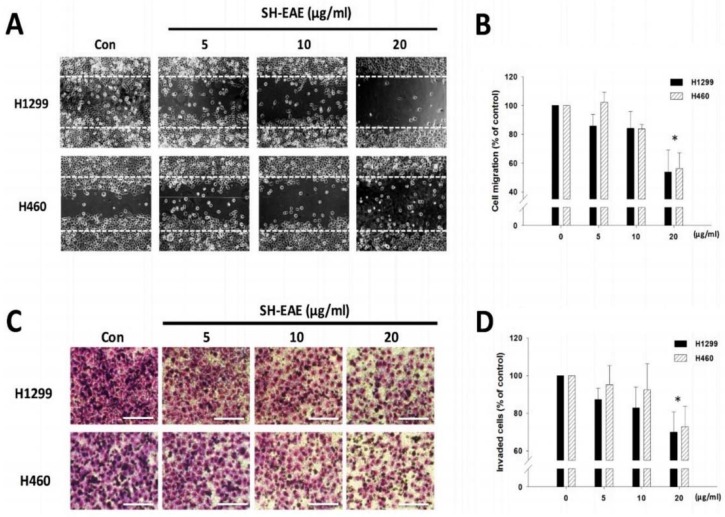
SH-EAE decreased H1299 and H460 cell migration and invasion in vitro. (**A**) Representative 4× magnification images at 16 h. H1299 and H460 cells in confluence were scratched and then treated with different concentration (5, 10, and 20 μg/mL) of SH-EAE for 16 h. The cells were fixed in 4% paraformaldehyde solution. The area between the two dotted lines indicates the wound width of SH-EAE (20 μg/mL)-treated cells at 16 h after scratching. (**B**) The quantifications of the regions of the cell during migration were analyzed using a software “TScratch”. Data represent the mean ± SD of three independent experiments. (* *p* < 0.05). (**C**) Cell invasion was determined using ThinCert™ cell culture transwell inserts, showing representative images of the bottom surface of the transwell membrane in H1299 and H460 cells. Scale bar indicates 1000 μm. (**D**) The number of invaded cells in four random microscopic fields (×200) was counted for each group. Data are shown as means ± SD of three independent experiments. (* *p* < 0.05).

We also assessed whether SH-EAE interferes with migration and invasion ability of NSCLC cells because the ER stress status has been reported to be associated with cancer metastasis [16]. Following treatment with SH-EAE, we found a decrease in migration ability of both H1299 and H460 cells (Figure 5A,B). When applied at 20 μg/mL, the ethyl acetate extract inhibited cell migration by almost 45%, as compared with vehicle controls. Besides, at a dose of 20 μg/mL, SH-EAE reduced cell invasion of H1299 and H460 cells by 35.9% and 27.3%, respectively (Figure 5C,D). Taken together, SH-EAE significantly suppresses the malignant phenotypes of NSCLC cells via modulating ER stress.

### 2.6. SH-EAE Reduced Phosphorylation and Expression of EGFR and VEGF Signaling in H1299 Cells

**Figure 6:**
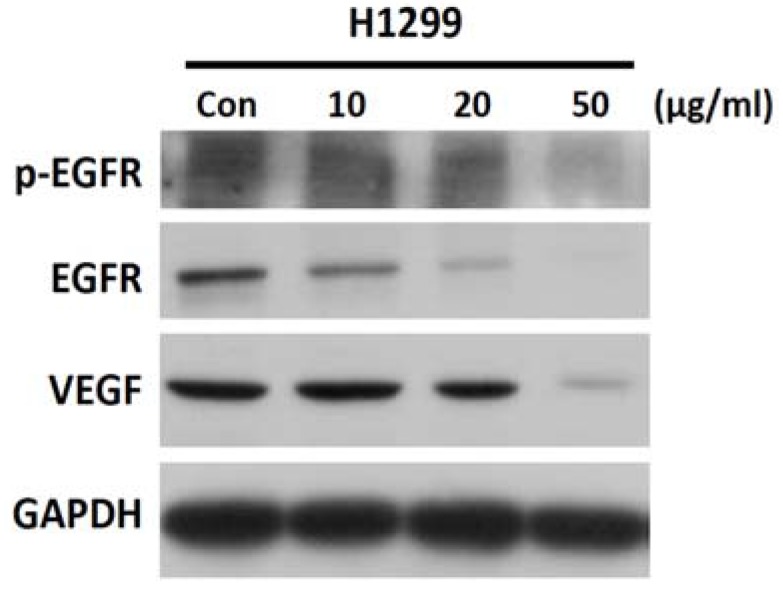
SH-EAE reduces the EGFR and VEGF signaling in NSCLC cells. Expression of phospho-EGFR (Tyr845), EGFR, and VEGF were analyzed by western blot in NSCLC cell line H1299. GAPDH was used as the loading control. Data are representative of three independent experiments.

Because treatment of NSCLC cells (H1299 and H460) with SH-EAE resulted in decreased cell proliferation and migration, SH-EAE treatment may attenuate the mitogenic signaling pathways in NSCLC cells. To test this possibility, H1299 cells were treated with increasing concentrations of SH-EAE for 48 h. Results show that SH-EAE reduced the phosphorylation and expression of epidermal growth factor receptor (EGFR), as well as reduced protein levels of the vascular endothelial growth factor (VEGF) (Figure 6). These findings uncover an additional pathway by which SH-EAE suppresses malignancy of NSCLC cells.

### 2.7. SH-EAE Was Not Poisonous Enough to Induce DNA Damage and Apoptosis in NSCLC Cells

**Figure 7:**
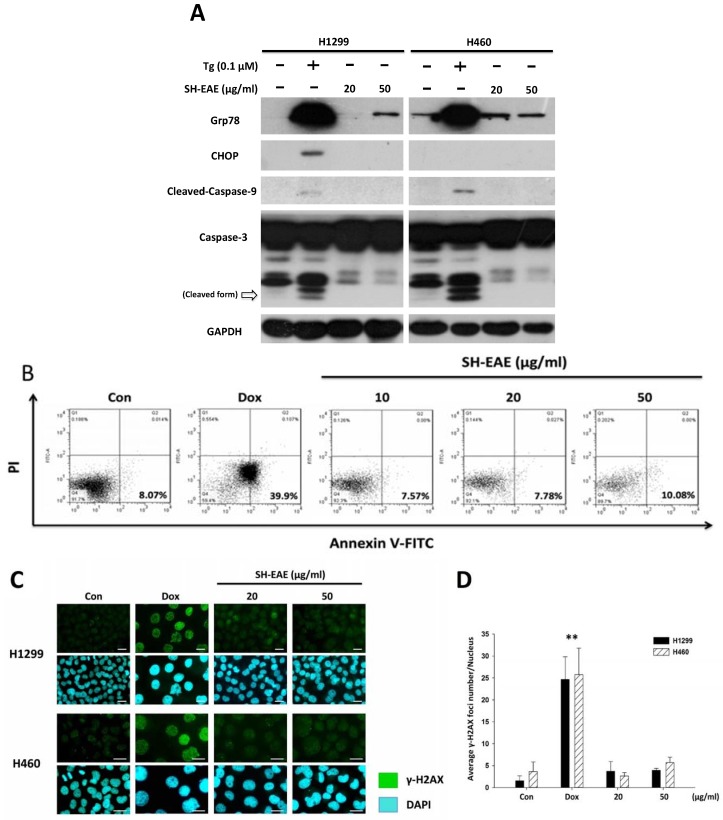
SH-EAE regulates UPR signaling but does not induce apoptosis. (**A**) Western blotting demonstrates relative protein levels of UPR components, including Grp78, CHOP, and proapoptotic proteins, such as caspase-9 and caspase-3, in NSCLC cells. Different from the Tg treatment. The increased levels of Grp78 in the SH-EAE treatment groups were obviously lower than the Tg group, and SH-EAE did not induce CHOP expression compared to Tg. The cleaved (active) form of caspase-3 and -9 were only induced in the Tg treatment. GAPDH serves as loading control. One of the two independent experiments is shown. (**B**) Annexin V-PI staining was performed to analyze apoptotic cell populations. 2 × 10^5^ cells were seeded into a six-well plate and treated with different concentrations (10, 20, and 50 μg/mL) and Dox (2 μM) for 48 h. Data are presented as dot plots (Annexin V-FITC on the *x*-axis; PI on the *y*-axis). The cell numbers in the four quadrants represent the percentage of viable (lower left), necrotic (upper left), early apoptotic (lower right), and late apoptotic (upper right) cells determined by using a BD Accuri™ C6 flow cytometer. Data are representative of three independent experiments. (**C**) A gallery of representative images of γ-H2AX foci was shown. Both H1299 and H460 cells were treated with SH-EAE (20 and 50 μg/mL) and Dox (2 μM) for 48 h. Cells were fixed and stained by immunofluorescence with anti-γ-H2AX (FITC, green). Nuclei were counterstained with DAPI. Representative images of H1299 and H460 cells obtained from at least three independent experiments for each condition are shown. ■ γ-H2AX; ■ DAPI. Scale bar indicates 50 μm. (**D**) Quantification of the numbers of γ-H2AX foci per nucleus after treatment with Dox or SH-EAE. Dox served as a positive control for DNA damage. The average numbers of γ-H2AX foci per cells treated with SH-EAE (20 and 50 μg/mL) were insignificant at 48 h as compared with untreated control cells. More than 50 cells were randomly analyzed using a software “ImageJ”. Data represent the mean ± SD of three independent experiments. (** *p* < 0.001).

Prolonged and severe ER stress has been reported to induce apoptosis via triggering a cascade of caspase activation. We therefore checked the apoptotic response following treatment with SH-EAE in H1299 and H460 cells. The active (cleaved) form of caspase-9 and caspase-3 have long been implicated as critical executioners during apoptosis, and both of them were markedly increased after treatment with ER stress inducer Tg for 48 h (Figure 7A). However, we did not observe any apoptotic response after 48 h of the SH-EAE treatment in both NSCLC cell lines (Figure 7A). Annexin V-Propidium Iodide (PI) staining also confirmed that SH-EAE did not induce apoptotic cell death in NSCLC cells compared to doxorubicin (Dox), a chemotherapeutic agent known to induce DNA damage (Figure 7B). In addition, the degree of DNA damage in NSCLC cells was determined by immunofluorescence staining of γ-H2AX, which is a prominent marker of DNA double-strand breaks [27]. As shown in Figure 7C,D, we observed that after 24 h of Dox treatment, the average number of γ-H2AX foci was significantly increased as compared to the control sample. In contrast, treatment with SH-EAE in NSCLC cells showed no increase in the average number of γ-H2AX foci, although there was slightly but not significantly higher foci occurring in SH-EAE-treated cells than in control cells. Based on these data, we conclude that SH-EAE is capable of impairing cell proliferation and migration, but fail to induce apoptosis and DNA damage in NSCLC cells.

### 2.8. SH-EAE Antagonizes the Induction of CHOP Expression by Tg

**Figure 8:**
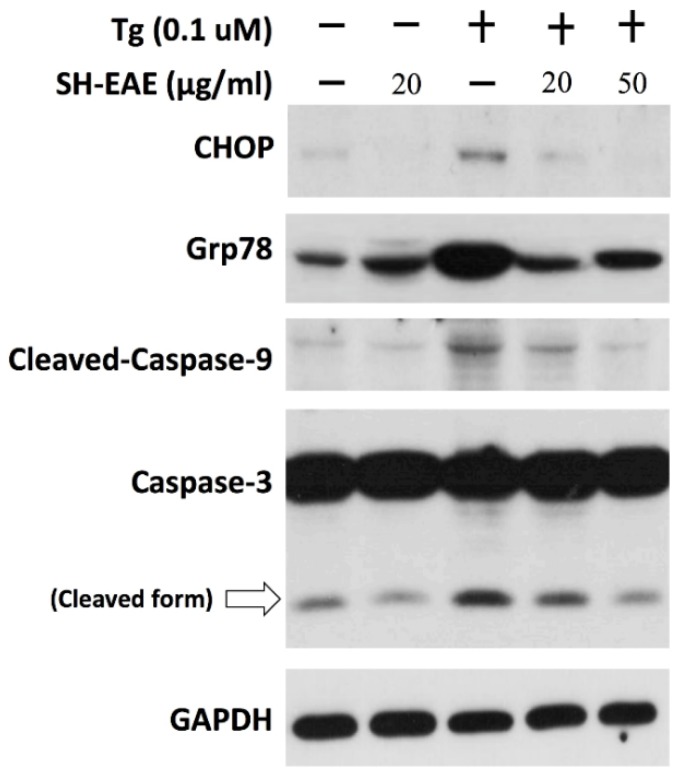
SH-EAE abrogated the Tg-mediated induction of Grp78 and CHOP expression. H1299 cells were pretreated with SH-EAE for 10 h and further treated with Tg for another 12 h. Protein lysates were subjected to SDS-PAGE followed by immunoblotting using antibodies against Grp78, CHOP, cleaved-caspase-9 and caspase-3. H1299 cells pretreated with 20 and 50 μg/mL SH-EAE were significantly inhibited by Tg-induced CHOP expression. α-tubulin served as the loading control.

SH-EAE acting through the inhibition of UPR signaling was confirmed by immunoblot analysis using pretreatment of H1299 cells with SH-EAE, being further treated with Tg, which served as an ER stress inducer. The protein expression of C/EBP homologous protein (CHOP) as an important transcription factor activated by the PERK pathway, was absent in untreated H1299 cells or cells treated with SH-EAE alone (Figure 8), and it was induced significantly in H1299 cells treated with 0.1 μM Tg alone. However, pretreatment with H1299 cells with 20 and 50 μg/mL SH-EAE, gradually inhibited the induction of Grp78 and CHOP expression evoked by Tg (Figure 8). Besides, our data also demonstrated that pretreatment with 20 and 50 μg/mL SH-EAE suppressed Tg-induced cleavage of caspase-3 and -9 in a dose dependent manner (Figure 8). These findings suggest that SH-EAE has an antagonistic effect on UPR signaling outputs in NSCLC cells.

### 2.9. SH-EAE Attenuated the Angiogenesis in Zebrafish Model

**Figure 9:**
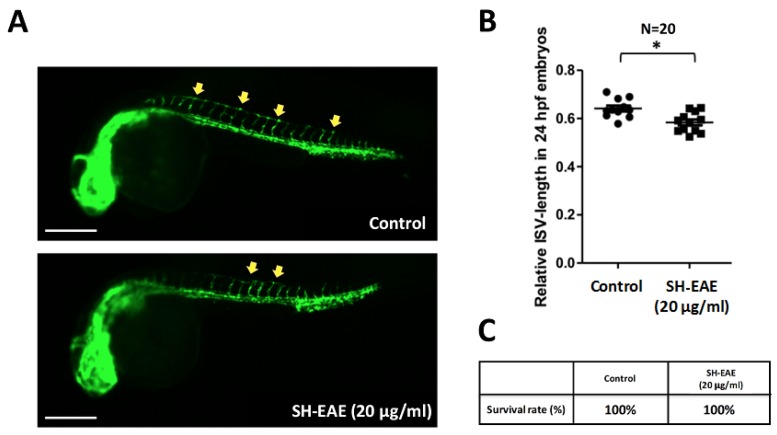
SH-EAE decreased angiogenesis in the zebrafish model. (**A**) The image is representative of 20 individual zebrafish larvae *Tg(flk1:EGFP)* under a fluorescence light microscope after exposure to SH-EAE (20 µg/mL) or vehicle control for 14 h. The upper image is untreated larvae as the control group, and the lower image is larva treated with SH-EAE (20 µg/mL). The yellow arrows depict the regions where fluorescent intersegmental vessels (ISVs) were obvious. Scale bars = 200 μm. (**B**) The formation of the ISVs in zebrafish larvae at 24 hpf. *n* = 20 embryos pooled from two independent experiments. Centerlines indicate median values. The *p*-value from the Mann–Whitney *U* test is less than 0.05 (* *p* < 0.05). (**C**) The effect of SH-EAE on the survival rate of zebrafish embryos. Data are expressed as percentages. *n* = 20 for each group.

Recently, IRE-1α and PERK have been postulated to play a role in the promotion of tumor angiogenesis [10,28,29]. To examine whether SH-EAE that dramatically suppressed the protein levels of IRE-1α and PERK can influence the angiogenesis in vivo, we tested the anti-angiogenic effects of SH-EAE with a zebrafish model. Zebrafish larvae derived from transgenic line *Tg(fli1:EGFP)* with fluorescent blood vessels were grown in embryo water (0.2 g/L of Instant Ocean Salt in distilled water) at 28 °C in 24-well plates with 10 larvae per well. Beginning at 10 h post fertilization (hpf), zebrafish larvae were exposed to 20 μg/mL of SH-EAE and assessed at 24 hpf. Treatment with SH-EAE significantly inhibited intersegmental vessels (ISVs) formation (Figure 9A,B). Continuous exposure to 20 μg/mL of SH-EAE by 48 hpf did not cause lethality and deleterious effect on the growth of zebrafish larvae. Consistent with our primary hypothesis, treatment of zebrafish larvae with SH-EAE resulted in a reduction of angiogenesis in the zebrafish model. This result was quite consistent with earlier studies showing reduced angiogenic ability in tumors that lack expression of UPR components [30].

## 3. Discussion

Provoking the ER stress is currently regarded as a promising therapeutic strategy in the treatment of cancer. In the present study, we found that ethyl acetate extract of *Scindapsus* cf. *hederaceus*, named as SH-EAE, has a great potential of targeting UPR-related proteins in NSCLC cells as evidenced by a substantial decrease in protein expression of ER stress sensors, including IRE-1α and PERK (Figure 2A). In addition, there is a decrease in Ero1-Lα, an essential oxidoreductase that is required for the formation of protein disulfide bonds in ER, reflecting the protein-folding burden imposed on ER. Some plants belonging to the family Araceae have been recognized to have anti-bacterial, anti-oxidant and anti-cancer activities. However, the association of ER stress with the extracts isolated from the *S.* cf. *hederaceus* has never been previously studied.

The present study shows that SH-EAE led to a dose-dependent increase in the protein expression of Grp78, which started to increase at 2 h and was maintained at a constant level over the following 6 to 24 h of 20 μg/mL SH-EAE treatment (Figure 2B). The increase in Grp78 is strikingly reminiscent of the UPR activation [31], implying that, within the first 4 h of SH-EAE treatment, the increased expression of Grp78 might serve as an early response that enables SH-EAE-treated cells with the ability to recover from ER stress. On the other hand, one of the three ER stress sensors, PERK, was found downregulated after 10 h of SH-EAE treatment, we therefore speculate that the loss of PERK may evoke a limiting circumstance for SH-EAE to continuously activate the UPR pathway and its downstream targets. Altogether, the above findings provide a possible explanation for why SH-EAE initially causes an increased expression of Grp78, but does not eventually reach the amounts of those Grp78 proteins induced by Tg (Figure 7A). Besides, the increased expression of Grp78 by SH-EAE is probably confined to the ER (Figure 2D). This result is much less consistent with the observed changes in cells treated with Tg or Tm, showing that Grp78 was not only prominent in ER, but also in the cytoplasm (Figure 2D). However, further research is needed to determine the exact cause of increased Grp78 in SH-EAE-treated cells.

Our study also sheds light on the interruption of UPR signaling by SH-EAE in NSCLC cell lines. The CHOP induction that occurred during severe ER stress was shown to be absent in SH-EAE-treated cells (Figure 8). Because CHOP/GADD15, a bZIP transcription factor belonging to the CCAAT/enhancer binding protein (C/EBP) family, is often associated with the apoptosis signal cascade [32]. We, therefore, checked for similarities between SH-EAE and Tg in terms of their effects on apoptosis and DNA damage. In Tg-treated cells, the cleaved forms of caspase-3 and -9 were predominantly observed. In contrast, SH-EAE treatment did not increase the expression of caspase-3 and -9 nor the expression of CHOP (Figure 7A). Similarly, SH-EAE did not induce apoptosis nor DNA damage as assessed by flow cytometric analysis of Annexin V/PI-stained cells (Figure 7B) and immunofluorescence detection of γ-H2AX foci (Figure 7C), respectively. Notably, the ability of the ER stress inducer Tg to induce the expression of CHOP and Grp78 was reduced by a pretreatment with SH-EAE (Figure 8), suggesting that the UPR is not fully activated by Tg.

Accumulation of misfolded proteins sometimes lead to the massive formation of vacuoles in the ER, making them deleterious to the cells. The present study also shows that SH-EAE was implicated in the formation of cytoplasmic vacuoles, which were likely derived from the ER (Figure 3). In contrast, both H1299 and H460 cells treated with Tg or Tm decreased the number of viable cells but did not undergo vacuolization (Figure 3A,B). These ER-derived vacuoles present in SH-EAE-treated cells are similar to what happens to unsolvable ER stress when cells were exposed to other natural product-derived compounds, such as Calphostin C [33], Celastrol [34], and Cyclosporine [35].

One possibility, raised by the work of Mimnaugh et al. [36], is that the inductive effect of SH-EAE on ER vacuolization may be due to interfering with the ability of ATPase valosin-containing protein (VCP), a known regulator involved in retrotransporting the misfolded proteins from ER into cytoplasm for ER-associated degradation (ERAD). A second possibility is that such form of vacuolization may be caused by the accumulation of unfolded proteins in ER or decreasing the export rate of ER proteins to Golgi. A third possibility, suggested by the early work of Wei-Jiunn Lee et al. [37], is that ER-derived vacuolization is driven by an increase in ROS production. The latter has been excluded from our study, because we noted that inclusion of NAC, a ROS scavenger, prevented neither the vacuole formation nor decreased the expression of Grp78, IRE-1α, and PERK in SH-EAE-treated NSCLC cells (Figure S1). Finally, there is no way to rule out the possibility that such vacuolization induced by SH-EAE was correlated with autophagy, which was also associated with vacuole formation and recycling of protein aggregates. However, the exact mechanism still needs further investigation.

Since physiological levels of ER stress are critical for maintaining cell homeostasis, we examined the effects of SH-EAE on NSCLC cell colony formation and migration ability. Firstly, we found that SH-EAE caused a colony formation loss in both H1299 and H460 lung cancer cell lines in a dose-dependent manner (Figure 4A,B). Notably, the growth rate of bronchial epithelial BEAS-2B cells did not seem to have been much affected in media with the same SH-EAE concentration as cancer cells (Figure 4A,B), suggesting that normal cells were poorly affected by SH-EAE treatment. In addition, there is a decrease in phosphorylation and total protein expression of EGFR by SH-EAE (Figure 6). Like other receptor tyrosine kinases, EGFR contributes to cancer progression through effects on cell proliferation, metastasis, angiogenesis, and inhibition of apoptosis [38,39]. Therefore, EGFR represents an ideal target for anticancer therapy. The observation that UPR is linked with the phosphorylation of EGFR is supported by a number of observations. One obvious example is that, under ER stress triggered by glucose deprivation, the ER resident chaperone Grp78 formed a complex with the underglycosylated EGFR and inhibited the translocation of EGFR to the cell surface, thus downregulating the EGF signaling pathway [40]. This is consistent with another study, which reported that treatment with EGF led to the activation of the PERK arm of the UPR, which especially increases the eIF2α phosphorylation and ATF4 protein expression [41]. Moreover, blocking the IRE1α-XBP1 axis of the UPR reduced the EGF-stimulated cell proliferation [41]. These studies provide convincing evidence that the cooperative signaling between UPR and EGFR is critical in regulating cancer cell proliferation.

Since angiogenesis is known as a prognostic marker in cancer, we further examined the protein expression of VEGF, together with in vivo anti-angiogenesis activity in SH-EAE-exposed zebrafish larvae. It was found that the expression of VEGF was significantly decreased in H1299 cells treated with 50 μg/mL SH-EAE (Figure 6). Moreover, in vivo experiments showed that administration of zebrafish with SH-EAE for a period displays potent anti-angiogenic activity without affecting the general morphology of developing zebrafish (Figure 9), suggesting that SH-EAE has no apparent side effects and it will benefit the applications of cancer prevention in future. Several studies focused on the role of UPR signaling in the promotion of angiogenesis. For example, a dysregulation of UPR is implicated in the pathogenesis of several vascular diseases, such as myocardial ischemia, atherosclerosis, hypertension, chronic heart failure, diabetes, diabetic retinopathy, and chronic renal failure, and some of these associations were thoroughly reviewed [42,43].

The effects of UPR on angiogenesis are not much different, depending upon the cell type and intensity of ER stress. Céline Philippe demonstrated that in the ischemic muscle, the translational activity for two angiogenesis-related genes were increased under hypoxic conditions in the ischemic muscle; including vascular endothelial growth factor (VEGF) and fibroblast growth factor 2 (FGF-2). This phenomenon becomes particularly evident under hypoxic stress, whereas the PERK arm of the UPR drives the translation of mRNAs encoding VEGF and FGF-2 [44]. It was also reported that VEGF-mediated cell survival in endothelial cells requires the activation of both the ATF6 and PERK pathways [45].

Besides, Benjamin Drogat demonstrated that the IRE-1α-XBP1 pathway contributes to ischemia-mediated angiogenesis and tumor growth in vivo [29]. Silencing of XBP1 led to reduced VEGF expression, suppressed endothelial cell proliferation, decreased myocardial capillary density, and exacerbation of isoproterenol-induced cardiac dysfunction [46]. Therefore, it was concluded that UPR is strongly correlated with angiogenesis. In our study, we demonstrated that decreased expression of PERK and IRE-1α in SH-EAE-treated NSCLC cells is accompanied by the reduction of angiogenesis, which is evidenced by a decreased expression of VEGF (Figure 6) and decreased development of intersegmental vessels (ISVs) in zebrafish larvae (Figure 9). Taken together, our data suggest the possibility that UPR may play a role in the SH-EAE-induced suppression of some angiogenic phenotypes.

ER stress is an intrinsic part of the deleterious process and needs to be mitigated for optimizing the protein quality. UPR is a cytoprotective response triggered by three ER stress sensors (PERK, IRE-1α, and ATF6), thereby restoring the ER homeostasis. This capacity of UPR, however, provides a tremendous contribution for tumorigenesis. Accordingly, a genetic deletion of one of the ER stress sensors, PERK, in embryonic stem cells renders cells prone to accumulate the misfolded proteins in ER and impairs their tolerance to ER stress [47]. A similar feature in the cancer-specific activation of UPR was observed during human histological analysis. Tissue samples from patients with advanced NSCLC tumors showed elevated levels of UPR-related proteins [48]. Based on this evidence, one can speculate that, in cancers, a blockage of UPR activation by pharmacological interventions results in abnormally elevated protein synthesis and a higher degree of ER stress.

Currently, the therapeutic potential of inducing ER stress in cancer can be divided into two groups: (1) induction of protein overloads in ER to hyper-activate UPR; (2) blockage of UPR adaptive pathways for rendering the cells intolerant to ER stress. The first group of agents, such as Bortezomib, Eeyarestatin I, 17AAG, and radicicol, trigger all three branches of the UPR by inhibiting the degradation of misfolded proteins and/or interfering with nascent protein folding inside the ER. Agents belonging to the second group, including GSK2656157, Irestatin, and Versipelostatin, mainly block the activation of UPR through inhibiting the kinase activity of PERK, the endonuclease activity of IRE-1α and the induction of Grp78 [49,50].

## 4. Experimental Section

### 4.1. Reagents and Antibodies

Thapsigargin (B6614) and tunicamycin (B7417) were both purchased from Apexbio Technology (Houston, TX, USA). Thapsigargin (Tg) and tunicamycin (Tm) were dissolved in dimethyl sulfoxide (DMSO) as 1 mM and 5 mg/mL stocks, respectively, and stored at −20 °C. Antibodies against the following proteins were used in this study: Grp78/Bip (#3177), Calnexin (#2679), Ero1-Lα (#3264), IRE1α (#3294), CHOP (#2895), PERK (#5683), PDI (#3501), cleaved caspase-9 (#9501S), Phospho(Tyr845)-EGFR (#4267) were from Cell Signaling (Beverly, MA, USA); HSP60 (GTX110089), GAPDH (GTX627408), α-Tubulin (GTX628820) were from GeneTex (Irvine, CA, USA). Antibody to caspase-3 (IMG-144A) was from IMGENEX (San Diego, CA, USA). Antibodies to ATF6 (ab135707) and EGFR (ab52894) were from Abcam (Cambridge, MA, USA). Antibodies to phospho-Histone H2A.X (γH2AX) (sc-101696) and phosphor-PERK (sc-32577) were from Santa Cruz Biotechnology (Santa Cruz, CA, USA). Antibody to VEGF (ABS82) was from Millipore (Billerica, MA, USA). Secondary antibodies were purchased from Leadgene Biomedical (#20102 and #20202, Tainan, Taiwan).

### 4.2. Preparation of the Extract of Scindapsus cf. hederaceus

**Figure 10:**
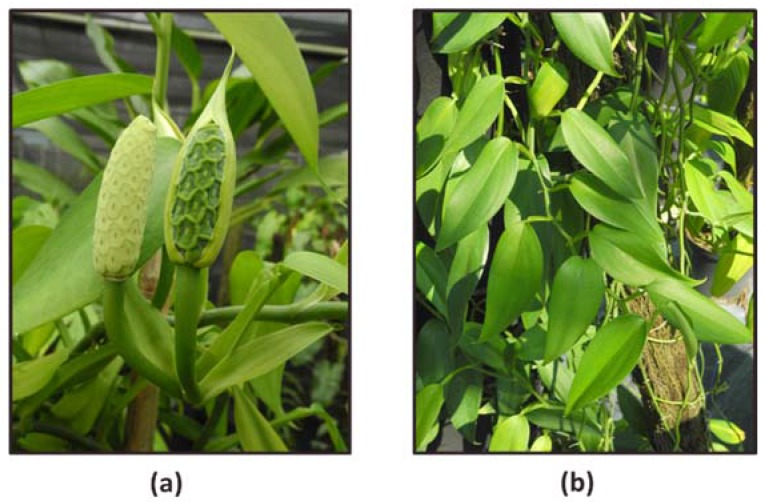
(**a**) The buds of *Scindapsus* cf. *hederaceus*; (**b**) the phyllotaxy, the arrangement of leaves on the stem of *Scindapsus* cf. *hederaceus*. Photos taken by Dr. Cecilia Koo Botanic Conservation Center (KBCC), Pingtung, Taiwan.

Plant Material: The specimens of *Scindapsus* cf. *hederaceus* were collected in Dr. Cecilia Koo Botanic Conservation Center, Kaohsiung County, Taiwan, in August, 2014. A voucher specimen (code no. KMU-NP067) was deposited in the Graduate Institute of Natural Products, College of Pharmacy, Kaohsiung Medical University, Kaohsiung, Taiwan (Figure 10).

Extraction and Partition: The fresh plant materials of *Scindapsus* cf. *hederaceus* (200 g) were extracted with methanol at room temperature and then concentrated under reduced pressure. The crude extract (1.7 g) was partitioned between ethyl acetate and water (1:1) to yield an ethyl acetate layer (0.6 g).

### 4.3. Cell Culture

Two NSCLC cell lines H1299 and H460, and the human bronchial epithelial cell line, BEAS-2B, were obtained from American Type Culture Collection (ATCC, Manassas, VA, USA). All cell lines studied were maintained in DMEM/F12 (Gibco, Grand Island, NY, USA) supplemented with 2 mM glutamine, 8% fetal bovine serum (FBS) and 0.5% penicillin/streptomycin solution (Mediatech, Inc., Herndon, VA, USA) at 37 °C in a humidified atmosphere of 5% CO_2_. Cell lines used in the present study were checked using a PCR-based assay and found to be free of mycoplasma contamination.

### 4.4. Colony Formation Assay

To determine the long-term effect of *Scindapsus* cf. *hederaceus* crude extract (SH-EAE) on the proliferative ability of a single cell to grow into an individual colony, the colony formation assay was performed. Briefly, H1299, H460, and BEAS-2B cells were seeded into 6-well plates at a density of 500 cells/well in triplicate. Cells were continuously exposed to vehicle or the indicated concentrations of SH-EAE for 14 days. Afterwards, cell colonies were fixed with 4% paraformaldehyde for 10 min and then stained overnight with 0.1% *w*/*v* Giemsa stain (Merck, Darmstadt, Germany) for overnight. The areas of these colonies were measured by the software Image-Pro (Media Cybernetics, Rockville, MD, USA).

### 4.5. Wound Healing Assay

Cells were seeded and grown in a 12-well plate until forming a full monolayer, which was then scratched by a pipet tip to create a straight wound. At the same time, cells were treated with indicated concentrations of SH-EAE. After 16 h of healing, the wound gaps were captured and analyzed by freeware “TScratch” (http://www.cse-lab.ethz.ch).

### 4.6. Transwell Invasion Assay

Cells were suspended in serum-free medium at a density of 5 × 10^5^ cells/mL. 200 μL of cell suspension was added to the apical compartment of the transwell inserts with 8-μm filter pores (ThinCerts™-TC Inserts, 24-well). The lower compartment was filled with 8% FBS-containing medium in the presence or absence of graded concentrations of SH-EAE. After 18 h of incubation, the non-invaded cells on the upper surface were gently wiped away using a cotton swab, and the invaded cells attached to the lower surface of the filter were fixed and stained with Giemsa stain (Merck, Darmstadt, Germany). Stained cells from the five randomly selected fields of view in each insert were counted under an inverted microscope (TE2000-U; Nikon, Tokyo, Japan) equipped with NIS-Elements Software (Nikon, Tokyo, Japan).

### 4.7. Western Blotting Assay

Whole-cell lysates were prepared on ice with RIPA buffer containing 50 mM Tris-HCl, pH 7.5, 0.15 M NaCl, 0.5% sodium deoxycholate, 1% NP-40 and 0.1% SDS. Equal amounts of total protein (30 μg protein per lane) were separated using SDS-polyacrylamide gel electrophoresis (SDS-PAGE) on 8–12% gels and transferred to a polyvinylidene fluoride (PVDF) membrane.

The PVDF membrane was blocked with Tris-based saline-0.5% Tween 20 (TBS-T) buffer containing 5% skim milk and then incubated with the primary and the corresponding secondary antibodies against specific proteins. After washing the membranes with TBS-T, the immune proteins were detected using a chemiluminescence kit (Advansta Corp., Menlo Park, CA, USA) following the manufacturer’s manual.

### 4.8. Quantitative Polymerase Chain Reaction Analysis

Total RNA was isolated from H1299 cells using the TRIzol reagent (Invitrogen, Carlsbad, CA, USA) following the manufacturer’s instruction. The quantity and quality of each RNA sample was measured with ND-1000 spectrophotometer (Nanodrop Technology, Wilmington, DE, USA), followed by conversion to cDNA using the high-capacity cDNA reverse transcription kit (Thermo Fisher Scientific-Applied Biosystems, Waltham, MA, USA) using 2 μg of mRNA to generate cDNA. Real-time PCR was carried out in a StepOne real time PCR system (Applied Biosystems, Foster City, CA, USA) using KAPA SYBR^®^ FAST ABI prism 2x qPCR master mix (Kapa Biosystems, Woburn, MA, USA) per the included protocol. Primer sequences for qPCR are listed at Table 1. GAPDH was used as a housekeeping gene based on our prior experience. The fold changes of mRNA expression were normalized to GAPDH reference to obtain the relative threshold cycle (C_t_).

### 4.9. Determination of ER Morphology

Analysis of the morphological changes of the ER in NSCLC cells after treatment with SH-EAE. Cells were incubated with ER-Tracker Red dye (Invitrogen, Carlsbad, CA, USA) at a final concentration of 1 μM in DMEM/F12 medium at 37 °C for 30 min. After washing thrice with PBS, cells were fixed and stained with nuclei staining dye 4′,6-diamidino-2-phenylindole (DAPI) for 5 min. Labeled fluorescent cells were further washed thrice by PBS before imaging them with fluorescence microscope (TE2000-U; Nikon, Tokyo, Japan).

### 4.10. Apoptosis Assessment

3 × 10^5^ cells were seeded in six well-plates and treated with the indicated concentrations of SH-EAE for 48 h. Doxorubicin (2 μM) was used as a positive control to induce cell apoptosis. After each treatment, both floating and adherent cells were collected, washed twice with PBS, and resuspended in Annexin V binding buffer. Cells were then stained with Annexin V-FITC and propidium iodide according to manufacturer instructions (Becton Dickinson, Bedford, MA, USA). Cells were analyzed using a FACSCalibur flow cytometer (Becton Dickinson, San Jose, CA, USA) and 10,000 events per sample were acquired. Data were analyzed using FlowJo software (Tree Star, San Carlos, CA, USA).

### 4.11. Immunofluorescence Staining

5 × 10^4^ cells were seeded on a glass slide, which has been steeped in 67% nitric acid, and treated with Tg (0.1 μM), Tm (0.5 μg/mL), or SH-EAE (20 and 50 μg/mL) for 48 h. At the end point of all treatments, cell staining with ER-Tracker, LysoTracker, and MitoTracker dye (Invitrogen, Carlsbad, CA, USA) was performed according to the manufacturer’s instruction, adding a final concentration of 1 μM for a period of 30 min (37 °C, 5% CO_2_). Cells were then fixed with 4% paraformaldehyde diluted in PBS for 5 min followed by permeabilization with 0.5% Triton x-100 in PBS for 10 min. Fixed cells were blocked with 1% bovine serum albumin (BSA) for 1 h, then incubated with primary antibody against Grp78/Bip (Cell Signaling, Danvers, MA, USA, #3177) at 4 °C overnight. After being washed thrice with 1% BSA, cells were stained with FITC-conjugated secondary antibodies (GeneTex, Irvine, CA, USA, GTX26816) at 4 °C for 1 h. At the last 5 min, nuclei were stained with 4′,6-diamidino-2-phenylindole (DAPI) for 5 min. Images were captured by fluorescence microscope (TE2000-U; Nikon, Tokyo, Japan).

### 4.12. In Vivo Assessment of Anti-Angiogenic Efficacy in Zebrafish

The use of a zebrafish model in cancer research is in accordance with the principles of 3Rs (reduction, replacement, and refinement), and approved by Institutional Animal Care and Use Committee of Kaohsiung Medical University Hospital, Kaohsiung, Taiwan (approval No. IACUC-105253). The transgenic fluorescent zebrafish *Tg(fli1:EGFP)* was obtained from the Taiwan Zebrafish Core Facility at Academia Sinica (TZCAS, Taipei, Taiwan). Briefly, zebrafish were raised and maintained in a 14:10 h light-dark cycle at 28 °C, and fed twice a day with frozen brine shrimp. Zebrafish embryos were generated by natural pairwise mating, and thousands of embryos were routinely produced by four to five pairs of adult zebrafish. Embryos were collected and kept in embryo water (0.2 g/L of sodium chloride in distilled water) at 28 °C while simultaneously removing the dead, unfertilized or abnormal embryos. 24 h post fertilization (hpf) embryos were then dechorionated with protease (1 mg/mL, Sigma, St. Louis, MO, USA) for 5 min at room temperature, washed several times with embryo water, and then distributed into a 24-well plate with 10 zebrafish larva in each well. These zebrafish larvae were incubated in embryo water at indicated concentrations of SH-EAE at 28 °C for 14 h. Afterwards, images of zebrafish expressing green fluorescent protein (GFP) specifically in endothelial cells were taken by stereo-fluorescent microscope (Leica ZF10) and then analyzed using the software ImageJ (National Institutes of Health, Bethesda, MD, USA). The anti-angiogenesis activity was determined by the fluorescent endpoint ISV-length detection method.

### 4.13. Statistical Analysis

Data are expressed as means ± SD (standard deviation). Significance of differences between various experimental and vehicle control groups were statistically analyzed by one-way of ANOVA using SigmaPlot v12 (Systat Software Inc., Point Richmond, CA, USA). A *p*-value of less than 0.05 was considered as statistically significant.

## 5. Conclusions

**Figure 11:**
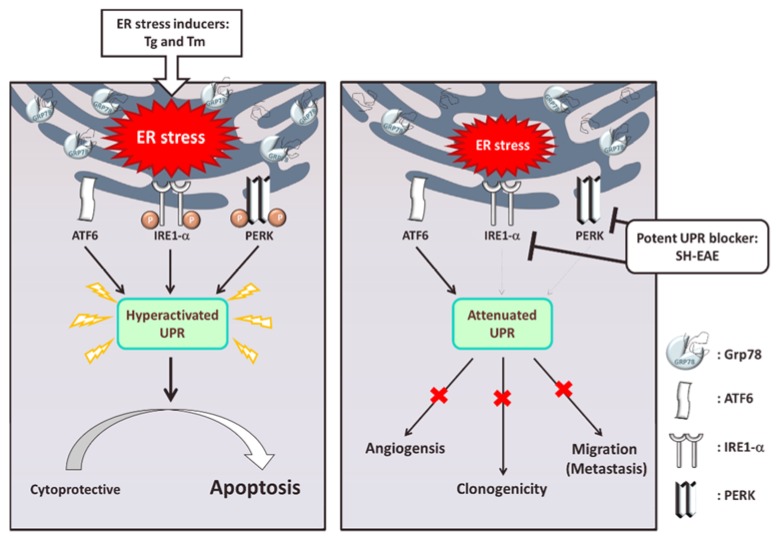
A proposed model of SH-EAE on anti-lung cancer effects through UPR modulation. Unfolded protein response (UPR) is a cytoprotective mechanism that alleviates the protein-folding burden in eukaryotic organisms. Both tunicamycin (Tm) and thapsigargin (Tg) cause a large accumulation of GRP78 chaperones in the ER lumen and the activation of all three sensors of ER stress ATF6, IRE1α, and PERK. This results in the hyperactivated UPR and the consequent growth arrest and apoptosis in cells. In contrast, SH-EAE selectively inhibited the clonogenicity, migration, and invasion in lung non-small cell lung cancer (NSCLC) cells through the attenuation of UPR by the downregulation of IRE1α and PERK.

In this study, we show that an ethyl acetate extract of *Scindapsus* cf. *hederaceus* (SH-EAE) has an ER stress response distinct from Tg and Tm. Given that SH-EAE possesses relatively mild toxicity and shows off-target effects on IRE-1α and PERK expression, we suggest that SH-EAE would attenuate UPR adaptive pathways for rendering the cells intolerant to ER stress (Figure 11). Our present work also implies that screening UPR regulators from plant extracts might be a useful methodology to identify novel ER stress inducers for cancer therapy or chemoprevention in the future.

## Figures and Tables

**Table 1:** Primers designed for real-time PCR in this study.

Genes	Primers
*GRP78*	F′: 5′-CAACCCCGAGAACACGGTC-3′
	R′: 5′-CTGCACAGACGGGTCATTC-3′
*ATF6*	F′: 5′-ATGTCTCCCCTTTCCTTATATGGT-3′
	R′: 5′-AAGGCTTGGGCTGAATTGAA-3′
*PERK*	F′: 5′-TGTCGCCAATGGGATAGTGACGAA-3′
	R′: 5′-AATCCGGCTCTCGTTTCCATGTCT-3′
*IRE1α*	F′: 5′-GGGAAATACTCTACCAGCCT-3′
	R′: 5′-GAAATCTCTCCAGCATCTTG-3′
*GAPDH*	F′: 5′-GTCTTCACCACCATGGAGAA-3′
	R′: 5′-ATGGCATGGACTGTGGTCAT-3′

## Supplementary Materials

Supplementary materials can be found at http://www.mdpi.com/1422-0067/19/7/1832/s1.
