# Supplementary material for: Ethyl Acetate Extract of Scindapsus cf. hederaceus Exerts the Inhibitory Bioactivity on Human Non-Small Cell Lung Cancer Cells through Modulating ER Stress
Source: Int J Mol Sci. 2018 Jun 21;19(7):1832. doi: 10.3390/ijms19071832 (PMC6073426; doi:10.3390/ijms19071832)
Supplement: Supplementary file 1 [file ijms-19-01832-s001.pdf]

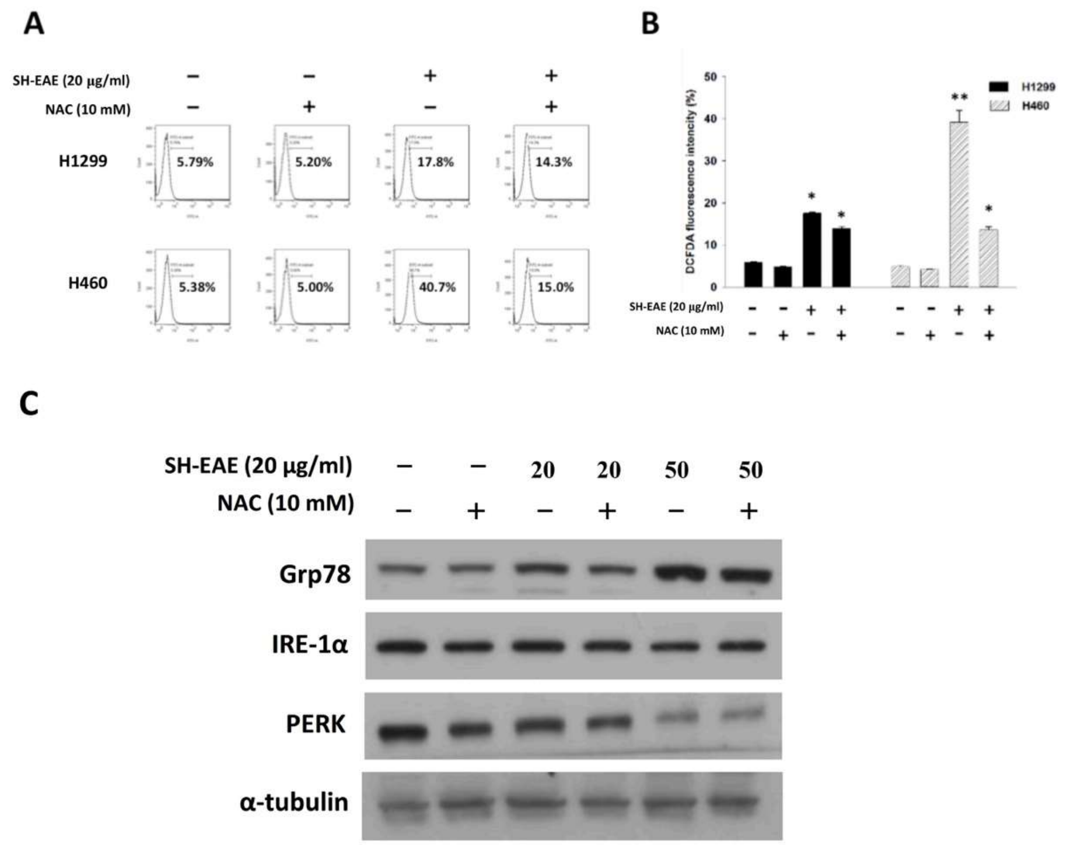

Figure S1. SH-EAE treatment enhanced the intracellular ROS levels in H1299 and H460 cells. (**A**) H1299 and H460 cells were pretreated with 10 mM of NAC for 1 h before treatment with 20 μg/mL of SH-EAE for another 12 h. The level of intracellular ROS was determined using the oxidant-sensing fluorescent probe DCF-DA, and the fluorescence was detected by flow cytometry (FACSCalibur, Becton Dickinson). Right shifts in fluorescent intensity indicate an increase of ROS. (**B**) the bar graph represents the average of three independent experiments (10000 cells were analyzed per experiment); data are shown as means ± SD (n = 3). (* $p < 0.05$; ** $p < 0.001$). (**C**) NAC did not reverse the SH-EAE-mediated downregulation of PERK expression while slightly decreasing the protein expression of Grp78 as compared with SH-EAE treatment alone. H1299 cells were pretreated with 10 mM of NAC for 1 h before treatment with 20 and 50 μg/mL of SH-EAE for another 12 h. Protein lysates were subjected to SDS-PAGE followed by immunoblotting using antibodies against Grp78, IRE-1α and PERK. α-tubulin served as the loading control.
